# Results of the inoperable and operable with aortic valve endocarditis

**DOI:** 10.3389/fcvm.2023.1296557

**Published:** 2024-01-16

**Authors:** Jing-bin Huang, Chang-chao Lu, Zhen-zong Du, Jian-rong Yang, Jun-jun Li

**Affiliations:** Department of Cardiothoracic Surgery, The People’s Hospital of Guangxi Zhuang Autonomous Region, and Guangxi Academy of Medical Sciences, Nanning, Guangxi, China

**Keywords:** aortic valve endocarditis, surgery, destruction of the aortic annulus, operable, inoperable

## Abstract

**Objectives:**

To evaluate the results of the inoperable and operable with aortic valve endocarditis, focus on risk factors, significance, and management of destruction of the aortic annulus in aortic valve endocarditis.

**Methods:**

The retrospective study was completed to investigate patients with aortic valve endocarditis undergoing cardiac surgery between January 2006 and November 2022 at our hospital.

**Results:**

512 patients were divided into group with destruction of the aortic annulus (*n* = 80) and without destruction of the aortic annulus (*n* = 432). There were 32 operative deaths (6.3%, 32/512). By univariate and multivariate analysis, destruction of the aortic annulus is found to be statistically significantly associated with in-hospital mortality (*P* < 0.001), prolonged mechanical ventilation time (mechanical ventilation time > 96 h, *P* = 0.018), early aortic paravalvular leak (*P* < 0.001), and 1-year mortality following cardiac surgery (*P* < 0.001), respectively.

**Conclusions:**

In our study, destruction of the aortic annulus increases mortality and health care costs. Optimization of pre-, peri-, and postoperative factors can reduce mortality and morbidity in aortic valve endocarditis. Aortic root replacement could be recommended as the best practice choice for aortic valve endocarditis with periannular abscess and destruction of the aortic annulus.

## 1 Introduction

Infective endocarditis (IE) is defined by a focus of infection within the heart and is a feared disease across the field of cardiology. Even at experienced centers, operations for IE remain associated with the highest mortality of any valve disease. Approximately half of all IE patients are identified as high-risk and undergo operative treatment. Destruction of the aortic annulus, which is caused by aortic periannular abscess or aortic root abscess, is a severe complication of aortic valve endocarditis. Surgical treatment of aortic valve endocarditis with destruction of the aortic annulus is a challenging issue with high mortality and morbidity rate in the current era (1–3).

In our cohort, the main reasons for perioperative death were paravalvular leaks and septic shock with consecutive multiorgan failure and cerebral hemorrhage. One of the most frequent determinants of paravalvular leaks is destruction of the aortic annulus in aortic valve endocarditis. However, only a few reports in the available literature evaluated the incidence and predictors for destruction of the aortic annulus in aortic valve endocarditis (4). We aimed to evaluate the results of the inoperable and operable with aortic valve endocarditis, focus on risk factors, significance, and management of destruction of the aortic annulus in aortic valve endocarditis.

We hypothesized that optimization of pre-, peri-, and postoperative factors can reduce mortality and morbidity in aortic valve endocarditis.

## 2 Patients and methods

### 2.1 Design

The retrospective study was completed to investigate patients with aortic valve endocarditis undergoing cardiac surgery between January 2006 and November 2022 at our hospital. Medical records were reviewed.

### 2.2 Diagnosis

Patients were diagnosed according to the modified Duke criteria (5). Surgical and pathological findings were reviewed to confirm the preoperative diagnosis.

### 2.3 Eligibility criteria

**Figure 1:**
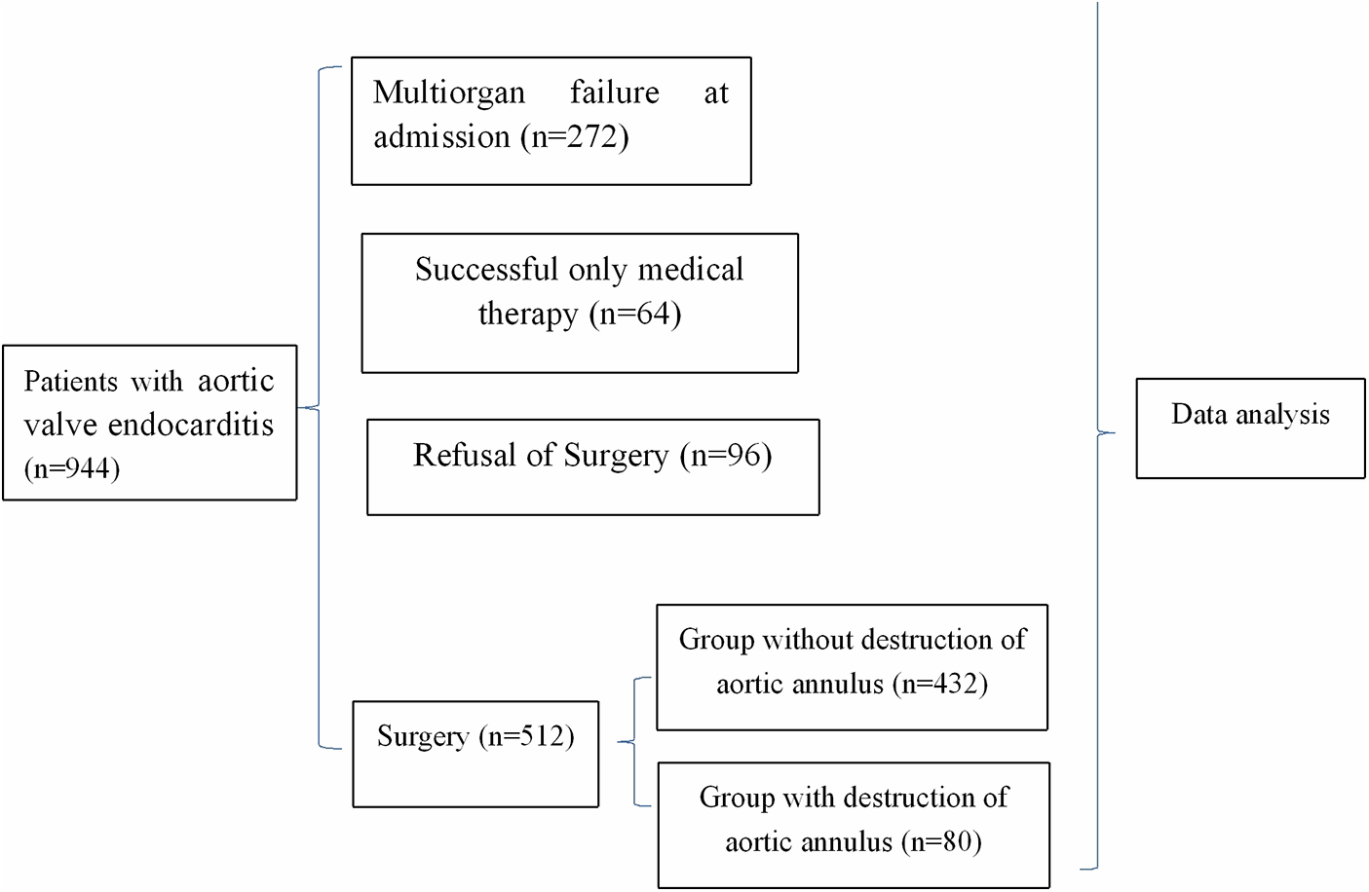
Flow chart of clinical trial. During the study period, 944 patients were diagnosed as aortic valve endocarditis, with 272 multiorgan failure at admission, 512 undergoing surgery, 64 receiving only medical therapy, and 96 refusal surgery.

Inclusion criteria included patients with aortic valve endocarditis undergoing cardiac surgery between January 2006 and November 2022 at our hospital. Exclusion criteria included patients without aortic valve endocarditis (Figure 1).

### 2.4 Surgical technique

Cardiac surgery for IE was indicated according to AATS guidelines for the management of IE. Radical debridement of all infected tissues and foreign material is followed by generous irrigation. Allografts are preferred for aortic root reconstruction in patients with annulus destruction (1).

### 2.5 Peri- and postoperative management

All patients were transferred to the intensive care unit after the operation. Anticoagulation management was conducted routinely. Transthoracic echocardiograph was performed postoperatively 1 to 7 days in intensive care unit.

### 2.6 Variables to be analyzed

Variables were evaluated (Supplementary Material).

Time between symptoms and admission was deﬁned as length from onset of symptoms to the date of admission.

Time between symptoms and surgery was deﬁned as length from onset of symptoms to the date of operation.

In-hospital mortality was defined as death within 30 days of the operation or during the same hospital admission.

In our study, serum creatinine was used as the diagnostic standard of acute renal injury. According to Kidney Disease Improving Global Outcomes (KDIGO) classification, if serum creatinine increases by ≥0.3 mg/dl (26.5 μmol/L) within 48 h, serum creatinine is 50% higher than the baseline within first seven days, or urine output is below 0.5 ml/kg/hour for six hours, the patient is considered to have acute renal injury (5).

Multiorgan failure (MOF) is regarded as a continuous process of varying levels of organ failure rather than an all-or-none event. Lungs, cardiovascular system, kidneys, liver, coagulation system, and central nervous system are regarded as “key organs” to characterize MOF (6).

Hepatic failure is defined as a severe liver injury, potentially reversible in nature and with onset of hepatic encephalopathy within 8 weeks of the first symptoms in the absence of pre-existing liver disease (7).

Respiratory failure is defined as a condition in which the respiratory system fails in one or both of its gas exchange functions, i.e., oxygenation of and/or elimination of carbon dioxide from mixed venous blood. It is deﬁned by an arterial oxygen tension (Pa, O2) of ≤8.0 kPa (60 mmHg), an arterial carbon dioxide tension (Pa, CO2) of ≥6.0 kPa (45 mmHg) or both (8).

### 2.7 Follow-up

All survivors discharged from hospital were monitored to the end date of the study. At the outpatient department, all patients were investigated with echocardiogram, electrocardiogram, and x-ray chest film, once every 3 to 12 months. At the last follow-up, the patients were contacted by telephone or micro-message or interviewed directly at the outpatient department.

### 2.8 Statistical analyses

Continuous variables are reported as means ± SE. A normality test was performed for all variables in the study by Kolmogorov-Smirnov test. Survival rates were estimated using the Kaplan-Meier method. The chi-square test, the Kruskal-Walls test or the Wilcoxon rank-sum test, as appropriate, was used to evaluate relationships between the preoperative variables, and selected intraoperative and postoperative variables. The relationships with perioperative risk factors were assessed by means of contingency table methods and logistic regression analysis. Survival analysis was performed by Kaplan–Meier analysis, and differences in survival between groups were examined with the log-rank test. Multivariate Cox proportional hazard models were used. *P* values less than 0.05 were statistically significant. All analyses were performed using IBM SPSS version 24.0 software (IBM SPSS Inc., USA).

### 2.9 Ethical approval

The experiment protocol for involving humans was in accordance to Helsinki Statement and national guidelines and was approved by the Medical Ethics Committee of The People's Hospital of Guangxi Zhuang Autonomous Region, and They gave the authors approval to waive the need for patient consent for publishing data in the study about the patients.

## 3 Results

### 3.1 Multiorgan failure at admission in aortic valve endocarditis

During the study period, 944 patients were diagnosed as aortic valve endocarditis, with 272 (28.8%, 272/944) excluded from surgery because of multiorgan failure at admission, 512 (54.2%, 512/944) undergoing surgery, 64 (6.8%, 64/944) receiving only medical therapy because of the absence of surgical indication, and 96 (10.2%, 96/944) being excluded from surgery, despite surgical indication, because of refusal of relatives (Figure 1).

Body weight (58.97 ± 0.28 vs. 55.14 ± 0.45 kg, *P* < 0.001), time between symptoms and admission (3.09 ± 0.13 vs. 2.32 ± 0.09 months, *P* < 0.001), vegetation length (14.42 ± 0.29 vs. 10.26 ± 0.23 mm, *P* < 0.001), aortic insufficiency (8.86 ± 0.53 vs. 5.68 ± 0.24 cm^2^, *P* < 0.001), and symptomatic neurological complications (47.1 vs. 11.9%, *P* < 0.001) in group of multiorgan failure at admission were significantly higher than those in group of non-multiorgan failure (Table 1).

**Table 1:** Multiorgan failure at admission in aortic valve endocarditis.

Variable	Group of multiorgan failure (*n* = 272)	Group of non-multiorgan failure (*n* = 672)	*P* value
Male	208 (76.5%)	512 (76.2%)	0.927
Age	40.29 ± 0.71	42.31 ± 0.6	0.054
Body weight	58.97 ± 0.28	55.14 ± 0.45	<0.001
Time between symptoms and admission	3.09 ± 0.13	2.32 ± 0.09	<0.001
Vegetation length	14.42 ± 0.29	10.26 ± 0.23	<0.001
Aortic insufficiency	8.86 ± 0.53	5.68 ± 0.24	<0.001
Symptomatic neurological complications	128 (47.1%)	80 (11.9%)	<0.001

### 3.2 Analysis of risk factors of multiorgan failure at admission in aortic valve endocarditis (*n* = 432)

Univariate analysis showed that factors are associated with multiorgan failure at admission, including body weight (*P* < 0.001), time between symptoms and admission (*P* = 0.037), vegetation length (*P* < 0.001), aortic insufficiency (*P* < 0.001), and symptomatic neurological complications (*P* < 0.001).

When they were included in multivariate analysis models, multivariate analyses also showed that factors are associated with multiorgan failure at admission, including body weight (*P* < 0.001), time between symptoms and admission (*P* < 0.001), vegetation length (*P* < 0.001), aortic insufficiency (*P* < 0.001), and symptomatic neurological complications (*P* < 0.001) (Table 2).

**Table 2:** Analysis of risk factors for multiorgan failure at admission in aortic valve endocarditis (*n* = 272).

Model	OR	95% CI	*P* value
Univariate analysis			
Body weight	0.960	0.945–0.975	<0.001
Time between symptoms and admission	0.868	0.817–0.922	<0.001
Vegetation length	0.891	0.869–0.914	<0.001
Aortic insufficiency	1.059	1.033–1.084	<0.001
Symptomatic neurological complications	6.578	4.713–9.181	<0.001
Multivariate analysis			
Body weight	0.955	0.937–0.974	<0.001
Time between symptoms and admission	0.833	0.782–0.888	<0.001
Vegetation length	0.890	0.867–0.914	<0.001
Aortic insufficiency	1.080	1.052–1.109	<0.001
Symptomatic neurological complications	7.610	5.145–11.256	<0.001

### 3.3 Successful only medical therapy in aortic valve endocarditis

64 patients were successfully treated only by medical therapy in aortic valve endocarditis. Time between symptoms and admission (1.0 ± 0.1 vs. 2.7 ± 0.2 months, *P* < 0.001) and vegetation length (5.4 ± 0.2 vs. 10.4 ± 0.9 mm, *P* < 0.001) in group of *successful only medical therapy* were significantly less than both in group of cardiac surgery (Table 3).

**Table 3:** Successful only medical therapy and refusal surgery in aortic valve endocarditis.

Variable	Group of only medical therapy (I) (*n* = 64)	Group of cardiac surgery (II) (*n* = 512)	Group of refusal surgery (III) (*n* = 96)	*P* value (I vs. II)	*P* value (I vs. III)	*P* value (II vs. III)
Male, *n*	48 (75%)	352 (71%)	71 (74%)	0.458	0.882	0.471
Age, years	42.4 ± 2.1	40.9 ± 0.7	49.3 ± 0.9	0.452	0.005	<0.001
Body weight, kg	55.6 ± 1.4	54.7 ± 0.5	57.0 ± 1.0	0.539	0.891	0.054
Time between symptoms and admission, months	0.54 ± 0.04	2.7 ± 0.2	1.65 ± 0.1	<0.001	<0.001	<0.001
Vegetation length, mm	5.0 ± 0.2	10.4 ± 0.9	13.83 ± 0.33	<0.001	<0.001	<0.001

### 3.4 Operative data

512 patients with aortic valve endocarditis undergoing cardiac surgery were divided into group with destruction of the aortic annulus (*n* = 80) and without destruction of the aortic annulus (*n* = 432). There were 32 operative deaths (6.3%, 32/512) (Tables 4, 5).

**Table 4:** Preoperative, operative and follow-up data (*n* = 512).

Variable	Total (*n* = 512)	Group with destruction of the aortic annulus (*n* = 80)	Group without destruction of the aortic annulus (*n* = 432)	*P* value
Preoperative				
Male, *n* (%)		(%)	(%)	
Age, years	40.63 ± 0.69	39.8 ± 1.2	40.78 ± 0.78	0.605
Weight, kg	54.69 ± 0.52	62.6 ± 0.58	53.22 ± 0.58	<0.001
Time between symptoms and surgery, months	2.64 ± 0.11	2.52 ± 0.28	2.66 ± 0.12	0.638
Vegetation length, mm	10.75 ± 0.3	13.2 ± 0.48	10.3 ± 0.33	<0.001
Preoperative left ventricular end diastolic dimension, mm	64.03 ± 0.38	70.6 ± 0.67	62.77 ± 0.40	<0.001
Preoperative left ventricular ejection fractions, %	60.32 ± 0.37	59.0 ± 0.81	60.58 ± 0.41	0.114
Preoperative aortic insufficiency, cm^2^	9.02 ± 0.28	12.20 ± 0.30	8.43 ± 0.32	<0.001
Serum creatinine before surgery, μmol/L	84.13 ± 1.26	105.6 ± 3.92	80.15 ± 1.21	<0.001
Operative				
In-hospital mortality, *n*	32 (6.3%)	18 (22.5%)	14 (3.2%)	<0.001
Aortic cross-clamp time, minutes	97.81 ± 1.56	122.8 ± 3.56	93.19 ± 1.63	<0.001
Cardiopulmonary bypass time, minutes	155.34 ± 2.27	209.8 ± 5.80	145.3 ± 2.15	<0.001
Mechanical ventilation time, hours	55.23 ± 2.42	73.2 ± 5.66	51.91 ± 2.64	0.001
ICU retention time, days	5.47 ± 0.13	7.0 ± 0.19	5.19 ± 0.15	<0.001
Hospitalized time postoperative, days	19.47 ± 0.38	21.2 ± 1.04	19.15 ± 0.4	0.047
Serum creatinine 24 h after surgery, μmol/L	98.59 ± 1.96	134.8 ± 7.27	91.89 ± 1.72	<0.001
Serum creatinine 48 h after surgery, μmol/L	116.94 ± 3.06	150.2 ± 11.57	110.78 ± 2.84	<0.001
Fluid balance on operation day, ml	−586.88 ± 36.21	−600.0 ± 48.78	−584.4 ± 42.0	0.876
Fluid balance on 1st day postoperative, ml	−637.50 ± 54.06	−440.0 ± 63.19	−674.1 ± 62.85	0.116
Fluid balance on 2nd day postoperative, ml	−446.88 ± 34.48	−200.0 ± 31.82	−492.6 ± 40.1	0.002
Chest drainage, ml	686.88 ± 15.41	786.0 ± 41.14	668.5 ± 16.48	0.006
Postoperative left ventricular end diastolic dimension, mm	49.61 ± 0.32	55.0 ± 0.64	48.58 ± 0.34	<0.001
Postoperative left ventricular ejection fractions, %	58.15 ± 0.34	55.4 ± 0.78	58.68 ± 0.37	<0.001
Fresh-frozen plasma, ml	722.81 ± 17.94	930.0 ± 36.53	684.4 ± 19.62	<0.001
Packed red cells, units	2.91 ± 0.12	4.20 ± 0.35	2.67 ± 0.12	<0.001
Follow-up	Total (*n* = 460)	Group with destruction of the aortic annulus (*n* = 60)	Group without destruction of the aortic annulus (*n* = 400)	*P* value
Length of follow-up, months	74.96 ± 2.44	36.5 ± 5.6	77.8 ± 2.6	<0.001
All-time mortality, *n*	79 (17.2%)	42 (70%)	37 (9.3%)	<0.001
All-time re-operation, *n*	41 (8.9%)	18 (30%)	23 (5.8%)	<0.001

ICU, intensive care unit.

**Table 5:** Operation in aortic valve endocarditis (*n* = 512).

Variable	Value
Operation	
Isolated aortic valve replacement, *n*	176/512 (34.4%)
Double valve operation, *n*	320/512 (62.5%)
Bentall + mitral valve replacement, *n*	16/512 (3.1%)
No root operation	432/512 (84.4%)
Aortic root operation	80 (15.6%)
6.0 Prolene sutures	15/80 (18.75%)
Patch reconstruction	49/80 (61.25%)
Aortic root replacement	16/80 (20%)

Weight (62.6 ± 0.58 vs. 53.22 ± 0.58 kg, *P* < 0.001), vegetation length (13.2 ± 0.48 vs. 10.3 ± 0.33 mm, *P* < 0.001), preoperative left ventricular end diastolic dimension (70.6 ± 0.67 vs. 62.77 ± 0.40 mm, *P* < 0.001), preoperative aortic insufficiency (12.2 ± 0.30 vs. 8.43 ± 0.32 cm^2^, *P* < 0.001), serum creatinine before surgery (105.6 ± 3.92 vs. 80.15 ± 1.21 μmol/L, *P* < 0.001) in group with destruction of the aortic annulus were significantly higher than those in group without destruction of the aortic annulus (Table 4).

### 3.5 Resource utilization

Aortic cross-clamp time (122.8 ± 3.56 vs. 93.19 ± 1.63 min, *P* < 0.001), cardiopulmonary bypass time (209.8 ± 5.8 vs. 145.3 ± 2.15 min, *P* < 0.001), mechanical ventilation time (73.2 ± 5.66 vs. 51.91 ± 2.64 h, *P* < 0.001), ICU retention time (7.0 ± 0.19 vs. 5.19 ± 0.15 days, *P* < 0.001), hospitalized time postoperative (21.2 ± 1.04 vs. 19.15 ± 0.4 days, *P* = 0.047), serum creatinine 24 h after surgery (134.8 ± 7.27 vs. 91.89 ± 1.72 μmol/L, *P* < 0.001), serum creatinine 48 h after surgery (150.2 ± 11.6 vs. 110.78 ± 2.84 μmol/L, *P* < 0.001), chest drainage (786.0 ± 41.14 vs. 668.5 ± 16.48 ml, *P* = 0.006), postoperative LVEDD (55.0 ± 0.64 vs. 48.58 ± 0.34 mm, *P* < 0.001), fresh-frozen plasma (930.0 ± 36.5 vs. 684.4 ± 19.62 ml, *P* < 0.001), Fresh-frozen plasma (930.0 ± 36.53 vs. 684.4 ± 19.62 ml, *P* < 0.001), and packed red cells (4.2 ± 0.35 vs. 2.67 ± 0.12 units, *P* < 0.001) in group with destruction of the aortic annulus were significantly higher than those in group without destruction of the aortic annulus (Table 4).

Fluid balance on 1st day postoperative (−440.0 ± 63.19 vs. −674.1 ± 62.85 ml, *P* < 0.001) and fluid balance on 2nd day (−200.0 ± 31.82 vs. −492.6 ± 40.1 ml, *P* < 0.001) postoperative in group with destruction of the aortic annulus was significantly less negative than that in group without destruction of the aortic annulus (Table 4).

The common early postoperative complications included acute renal injury (159/512, 31.1%), long-term intubation time >48 h (230/512, 44.9%), and multiorgan failure (52/512, 10.2%).

### 3.6 Analysis of risk factors for destruction of the aortic annulus

Univariate analysis of potential risk factors for destruction of the aortic annulus in patients with aortic valve endocarditis showed that numerous factors are associated with destruction of the aortic annulus, including body weight (*P* < 0.001), vegetation length (*P* < 0.001), preoperative aortic insufficiency (*P* < 0.001), preoperative left ventricular end diastolic dimension (*P* < 0.001), and serum creatinine before surgery (*P* < 0.001).

When they were included in multivariate analysis models, multivariate analyses also showed that numerous factors are associated with destruction of the aortic annulus, including body weight (*P* < 0.001), vegetation length (*P* < 0.001), preoperative aortic insufficiency (*P* < 0.001), preoperative left ventricular end diastolic dimension (*P* < 0.001), and serum creatinine before surgery (*P* < 0.001) (Table 6).

**Table 6:** Analysis of risk factors for destruction of the aortic annulus in aortic valve endocarditis.

Model	OR	95% CI	*P* value
Univariate analysis			
Body weight	0.898	0.868–0.928	<0.001
Vegetation length	0.942	0.911–0.974	<0.001
Preoperative aortic insufficiency	0.919	0.888–0.952	<0.001
Preoperative left ventricular end diastolic dimension	0.900	0.874–0.926	<0.001
Serum creatinine before surgery	0.970	0.962–0.979	<0.001
Multivariate analysis			
Body weight	0.897	0.862–0.933	<0.001
Vegetation length	0.926	0.887–0.967	0.001
Preoperative aortic regurgitation	0.934	0.897–0.972	0.001
Preoperative left ventricular end diastolic dimension	0.928	0.897–0.959	<0.001
Serum creatinine before surgery	0.976	0.966–0.987	<0.001

### 3.7 Analysis of significance of destruction of the aortic annulus in aortic valve endocarditis

Univariate and multivariate analysis of risk factors of in-hospital mortality, prolonged mechanical ventilation time (mechanical ventilation time >96 h), prolonged ICU retention time, early aortic paravalvular leak following cardiac surgery, and 1-year mortality following cardiac surgery in aortic valve endocarditis showed that destruction of the aortic annulus is statistically significantly associated with in-hospital mortality (*P* < 0.001), prolonged mechanical ventilation time (mechanical ventilation time >96 h, *P* = 0.018), early aortic paravalvular leak (*P* < 0.001), and 1-year mortality following cardiac surgery (*P* < 0.001), respectively (Table 7).

**Table 7:** Analysis of the significance of destruction of the aortic annulus in aortic valve endocarditis (*n* = 512).

Model	OR	95% CI	*P* value
Univariate analysis of risk factors for in-hospital mortality following cardiac surgery (*n* = 32)			
Destruction of the aortic annulus	6.500	3.097–13.641	<0.001
Multivariate analysis of risk factors for in-hospital mortality following cardiac surgery (*n* = 32)			
Destruction of the aortic annulus	5.847	2.767–12.355	<0.001
Univariate analysis of risk factors for prolonged mechanical ventilation time (mechanical ventilation time > 96 h, *n* = 128) following cardiac surgery			
Destruction of the aortic annulus	2.333	1.413–3.853	0.001
Multivariate analysis of risk factors for prolonged mechanical ventilation time (mechanical ventilation time > 96 h, *n* = 128) following cardiac surgery			
Destruction of the aortic annulus	1.890	1.117–3.917	0.018
Univariate analysis of risk factors for prolonged ICU retention time (ICU retention time > 7d, *n* = 176) following cardiac surgery			
Destruction of the aortic annulus	11.429	6.344–20.588	<0.001
Multivariate analysis of risk factors for prolonged ICU retention time (ICU retention time > 7d, *n* = 176) following cardiac surgery			
Destruction of the aortic annulus	9.956	5.413–18.313	<0.001
Univariate analysis of risk factors for early aortic paravalvular leak following cardiac surgery (*n* = 28)			
Destruction of the aortic annulus	7.448	4.228–12.936	<0.001
Multivariate analysis of risk factors of early aortic paravalvular leak following cardiac surgery (*n* = 28)			
Destruction of the aortic annulus	7.183	4.036–12.785	<0.001
Univariate analysis of risk factors for 1-year mortality following cardiac surgery (*n* = 46)			
Destruction of the aortic annulus	25.467	12.502–51.877	<0.001
Multivariate analysis of risk factors for 1-year mortality following cardiac surgery (*n* = 46)			
Destruction of the aortic annulus	43.316	12.77–146.91	<0.001

ICU, intensive care unit.

### 3.8 Follow-up results

**Figure 2:**
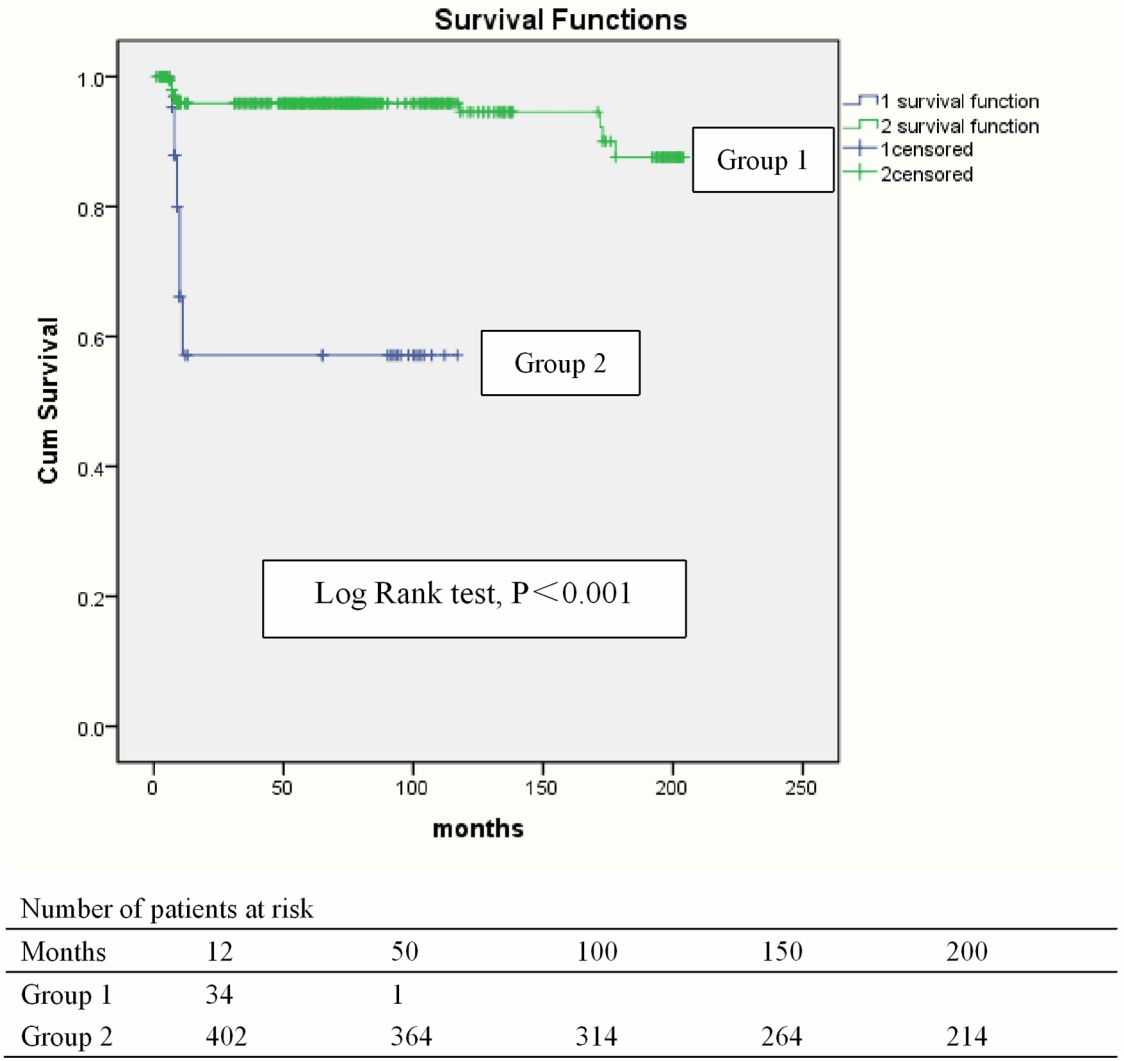
Kaplan–Meier curve for survival. Blue line, Group 1: Group with destruction of the aortic annulus. Green line, Group 2: Group without destruction of the aortic annulus.

480 survivors were discharged and 460 patients were monitored to the end date of the study and the follow-up was 95.8% (460/480) completed. The mean duration of follow-up was 74.96 ± 2.44 months. 55 deaths (55/460, 12.0%) occurred within 12 months after being discharged because of recurrence of IE and cerebral hemorrhage. The latest data of follow-up showed that 373 survivors were in NYHA class I (373/405, 92.1%) and 32 in class II (32/405, 7.9%). The presence of destruction of the aortic annulus in the setting of infective endocarditis significantly increased in-hospital mortality, and was also a significant risk factor for long-term survival. Univariate and multivariate analysis of Cox proportional hazard regression for all-time mortality indicated that numerous factors are associated with all-time mortality, including age (*P* < 0.001), body weight (*P* < 0.001), vegetation length (*P* < 0.001), preoperative aortic insufficiency (*P* < 0.001), ICU retention time (*P* < 0.001), serum creatinine 48 h after surgery (*P* < 0.001), postoperative left ventricular end diastolic dimension (*P* < 0.01), and packed red cells (*P* < 0.001) (Figure 2, Table 8).

**Table 8:** Cox proportional hazard regression for all-time mortality.

Model	OR	95% CI	*P* value
Univariate analysis			
Male	0.026	0.001–0.542	0.018
Age	0.951	0.924–0.979	0.001
Body weight	1.055	1.028–1.083	<0.001
Vegetation length	1.085	1.040–1.131	<0.001
Preoperative aortic insufficiency	1.041	1.004–1.079	0.029
ICU retention time	1.699	1.455–1.983	<0.001
Serum creatinine 48 h after surgery	1.301	1.170–1.458	<0.001
Postoperative left ventricular end diastolic dimension	1.041	1.010–1.073	0.004
Packed red cells	1.330	1.218–1.453	<0.001
Multivariate analysis			
Age	0.938	0.905–0.973	0.001
Body weight	1.043	1.020–1.067	<0.001
Vegetation length	1.407	1.226–1.616	<0.001
Preoperative aortic insufficiency	0.888	0.829–0.951	0.001
ICU retention time	1.477	1.214–1.798	<0.001
Serum creatinine 48 h after surgery	1.290	1.159–1.338	<0.001
Postoperative left ventricular end diastolic dimension	1.309	1.173–1.462	<0.001
Packed red cells	1.830	1.540–2.174	<0.001

ICU, intensive care unit.

## 4 Discussion

The present study aimed to evaluate the results of the inoperable and operable with aortic valve endocarditis, focus on risk factors, significance, and management of destruction of the aortic annulus in aortic valve endocarditis. We identified that destruction of the aortic annulus increases mortality and health care costs; optimization of pre-, peri-, and postoperative factors can reduce mortality and morbidity in aortic valve endocarditis; and aortic root replacement could be recommended as the best practice choice for aortic valve endocarditis with periannular abscess and destruction of the aortic annulus.

In our study, only 54.2% (512/944) patients with aortic valve endocarditis underwent surgery, and 28.8% (272/944) were excluded from surgery because of multiorgan failure at admission. Time between symptoms and admission is associated with multiorgan failure at admission. Vegetation length, aortic insufficiency, and symptomatic neurological complications are parameter of severity of disease. Vegetation length is associated with multiorgan failure at admission, destruction of the aortic annulus in aortic valve endocarditis and all-time mortality. Therefore, early diagnosis and treatment of aortic valve endocarditis are important, and early surgical intervention is advocated, as aortic valve endocarditis is a progressive and life-threatening disease. Reaching a rapid and accurate diagnosis in cases of aortic valve endocarditis is still a central challenge of the disease. Delayed diagnosis and initiation of therapy lead to complications and worse clinical outcomes. Because of lack of excellent network of primary, secondary, and tertiary prevention, these patients therefore present late in the hospital and are usually diagnosed and transferred to our tertiary hospital late. A conceptual framework comprising of required baseline information and requirements for executing primary, secondary, and tertiary prevention has been advocated as a best model for aortic valve endocarditis control.

Follow-up data about morbidity and mortality indicated that almost all the deaths occurred within 12 months of surgery, which means all patients discharged from hospital must be followed up closely. Vegetation length, preoperative aortic insufficiency, serum creatinine 48 h after surgery, postoperative left ventricular end diastolic dimension, and packed red cells are associated with all-time mortality, therefor, early diagnosis and initiation of therapy to avoid larger vegetation length and preoperative aortic insufficiency, and excellent protection for the kidney, heart, and blood have significant implication for long-term outcomes.

### 4.1 Destruction of the aortic annulus increases mortality and health care costs

The presence of a paravalvular abscess or destruction of aortic annulus is a crucial indication for surgical management due to its known association with high in-hospital mortality. Periannular abscess or destruction of aortic annulus occurs in more than 10% of patients with aortic valve endocarditis. The previous study indicated that about 22% of patients who suffered from aortic valve endocarditis had periannular abscess and destruction of aortic annulus. Another study reported periannular extension, including abscess, pseudoaneurysm and fistula accounted for 37% of left-sided endocarditis (4, 9–10). Delayed surgery may lead to further damage to the aortic root, with a higher risk of complications and a more complex surgical process. Despite progress in treatment, the associated incidence rate and mortality rates are still high, indicating the need for more effective interventions. Generally speaking, in these cases, the main goal of surgery is to thoroughly debride the infected tissue, reconstruct the excised area, and replace the aortic valve or aortic root. The choice of aortic valve replacement or aortic root replacement for annulus reconstruction depends on the degree of annulus destruction. Aortic root replacement surgery has been the main choice in recent studies (11). In our study, 15.6% (80/512) of patients with aortic valve endocarditis suffered from periannular abscess and destruction of aortic annulus. The presence of destruction of the aortic annulus in the setting of infective endocarditis significantly increased in-hospital and long-term mortality.

Complex infection of the aortic valve with aortic root involvement and destruction of aortic annulus remains a grave challenge to the cardiac surgeon. Patients frequently present profoundly unwell and extensive surgery may be required to correct the underlying anatomical deficits and provide source control in septic patients. Complex aortic valve endocarditis with aortic root involvement and destruction of aortic annulus continues to have a high in-hospital mortality rate of 10% to 40% (11–15). Improvement of surgical and perfusion techniques can decrease aortic cross-clamp time, cardiopulmonary bypass time, intubation time, ICU retention time, hospitalized time postoperative, paravalvular leak, chest drainage, fresh-frozen plasma, and packed red cells, therefor, contribute to better clinical outcomes.

### 4.2 Prevention of destruction of the aortic annulus

Preoperative LVEDD, vegetation length, preoperative aortic insufficiency, and serum creatinine before surgery are all parameter of severity of aortic valve endocarditis. Our present study showed that vegetation length and preoperative aortic insufficiency are associated with destruction of the aortic annulus in patients with aortic valve endocarditis. Therefore, early diagnosis and treatment of IE are important. Early surgical intervention is advocated, as aortic valve endocarditis is a progressive and life-threatening disease, and patients with a poor preoperative functional class are at the highest risk for in-hospital death. Early diagnosis and treatment maybe prevent the development of periannular abscess and destruction of aortic annulus.

### 4.3 Management of destruction of the aortic annulus

Surgical treatment of aortic valve endocarditis with aortic periannular abscess and destruction of the aortic annulus is still a challenging issue with high mortality and morbidity rate in the current era. Surgical method such as aortic valve replacement and aortic root replacement or reconstruction depend on the integrity of aortic annulus. According to the guideline of the Society of Thoracic Surgeons Clinical Practice, if the aortic annulus is healthy and strong enough for suture, it is reasonable to use a mechanical or stented tissue valve following radical debridement completed. If the aortic annulus is disrupted, aortic root replacement or reconstruction is usually the preferred choice of surgery (16, 17).

There is more and more evidence to indicate that aortic root replacement is associated with advantageous reoperation rates and rate of early paravalvular leak, a hypothesis that is also intuitive and backed by well-established surgical principles. Patients treated with an aortic root replacement were at a lower risk of reoperation and rate of early paravalvular leak at 1-year follow-up.

Destruction of the aortic annulus is associated with early aortic paravalvular leak, in-hospital mortality, and 1-year mortality. The main reasons of in-hospital mortality were early paravalvular leak and sepsis. Early paravalvular leak occurred mainly in patients with destruction of the aortic annulus treated with 6.0 Prolene sutures and patch reconstruction (early aortic paravalvular leak, 29.7%, 19/64). A paravalvular leak (PVL) is an incomplete apposition of a heart valve prosthesis on the native valve annulus, with a residual leakage between the artificial valve and the patient's native valve ring. This complication is the most common form of nonstructural valve dyfunction observed after a heart valve replacement. In Bentall procedure, a self-assembled composite graft allows safe proximal fixation of the conduit in patients with destroyed aortic annulus, resulting in sufficient proximal anastomosis and a very low incidence of aorta-related reoperations.Aortic paravalvular leak generally do not occur in aortic root replacement (Bentall procedure). It is reportet that modified Bentall procedure could have a role in effectively reducing paravalvular leak in aortic regurgitation related to Behcet's disease (18–21).

Aortic root replacement accomplishes more comprehensive removal of infected tissue and reconstruction of cardiac morphology compared with aortic valve replacement and therefor can attain lower rates of reinfection and graft deterioration. The aortic root replacement technique is related to an advantageous postoperative profile compared with aortic valve replacement. Aortic root replacement could be recommended as the best practice choice for aortic valve endocarditis with periannular abscess and destruction of the aortic annulus (22–25).

### 4.4 Perspectives

How to prevent multiorgan failure at admission and optimally treat destruction of the aortic annulus in aortic valve endocarditis remains grave challenge to us. Network of primary, secondary, and tertiary prevention and early diagnosis and treatment of aortic valve endocarditis will contribute to better short and long-term outcomes.

### 4.5 Strengths and limitations of the study

Our study for the first time clarified the risk factors, significance, and management of destruction of the aortic annulus in aortic valve endocarditis, adding value to the existing literature. Limitations of the present study include its retrospective design. There may be a selection bias because of the retrospective nature of the study and our hospital as a tertiary referral center. Well-designed research such as prospective cohort studies are needed and programs aiming at the reduction of in-hospital morbidity and mortality caused by aortic valve endocarditis are encouraged. The results and analysis of patients operated for aortic valve endocarditis is also very conflicting. Most of the risk factor CI interval is <1 suggesting they are protective. This needs to be re-evaluated. The diagnose of aortic root destruction was made during cardiac surgery, and the incidence of aortic root destruction in patients who had multiorgan failure at presentation and patients who refused surgery remained unknown.

## 5 Conclusions

In our study, destruction of the aortic annulus increases mortality and health care costs. Optimization of pre-, peri-, and post-operative factors can reduce mortality and morbidity in aortic valve endocarditis. Aortic root replacement could be recommended as the best practice choice for aortic valve endocarditis with periannular abscess and destruction of the aortic annulus.

## Data Availability

The raw data supporting the conclusions of this article will be made available by the authors, without undue reservation.
