# Supplementary material for: Results of the inoperable and operable with aortic valve endocarditis
Source: Front Cardiovasc Med. 2024 Jan 16;10:1296557. doi: 10.3389/fcvm.2023.1296557 (PMC10824924; doi:10.3389/fcvm.2023.1296557)
Supplement: Supplementary file 1 [file Table1.docx]

Table 1. Multiorgan failure at admission in aortic valve endocarditis

| Variable | Group of multiorgan failure (n=272) | Group of non-multiorgan failure (n=672) | P value |
| --- | --- | --- | --- |
| Male | 208 (76.5%) | 512 (76.2%) | 0.927 |
| Age | 40.29±0.71 | 42.31±0.6 | 0.054 |
| Body weight | 58.97±0.28 | 55.14±0.45 | ＜0.001 |
| Time between symptoms and admission | 3.09±0.13 | 2.32±0.09 | ＜0.001 |
| Vegetation length | 14.42±0.29 | 10.26±0.23 | ＜0.001 |
| Aortic insufficiency | 8.86±0.53 | 5.68±0.24 | ＜0.001 |
| Symptomatic neurological complications | 128 (47.1%) | 80 (11.9%) | ＜0.001 |
