# Supplementary material for: Results of the inoperable and operable with aortic valve endocarditis
Source: Front Cardiovasc Med. 2024 Jan 16;10:1296557. doi: 10.3389/fcvm.2023.1296557 (PMC10824924; doi:10.3389/fcvm.2023.1296557)
Supplement: Supplementary file 2 [file Table2.docx]

Table 2. Analysis of risk factors for multiorgan failure at admission in aortic valve endocarditis (n=272)

| Model | OR | 95% CI | P value |
| --- | --- | --- | --- |
| Univariate analysis | | | |
| Body weight | 0.960 | 0.945-0.975 | ＜0.001 |
| Time between symptoms and admission | 0.868 | 0.817-0.922 | ＜0.001 |
| Vegetation length | 0.891 | 0.869-0.914 | ＜0.001 |
| Aortic insufficiency | 1.059 | 1.033-1.084 | ＜0.001 |
| Symptomatic neurological complications | 6.578 | 4.713-9.181 | ＜0.001 |
| Multivariate analysis | | | |
| Body weight | 0.955 | 0.937-0.974 | ＜0.001 |
| Time between symptoms and admission | 0.833 | 0.782-0.888 | ＜0.001 |
| Vegetation length | 0.890 | 0.867-0.914 | ＜0.001 |
| Aortic insufficiency | 1.080 | 1.052-1.109 | ＜0.001 |
| Symptomatic neurological complications | 7.610 | 5.145-11.256 | ＜0.001 |
