# Supplementary material for: Results of the inoperable and operable with aortic valve endocarditis
Source: Front Cardiovasc Med. 2024 Jan 16;10:1296557. doi: 10.3389/fcvm.2023.1296557 (PMC10824924; doi:10.3389/fcvm.2023.1296557)
Supplement: Supplementary file 3 [file Table3.docx]

Table 3. Successful only medical therapy and refusal surgery in aortic valve endocarditis

| Variable | Group of only medical therapy (I) (n=64) | Group of cardiac surgery (II) (n=512) | Group of refusal surgery (III) (n=96) | P value  (I VS II) | P value  (I VS III) | P value  (II VS III) |
| --- | --- | --- | --- | --- | --- | --- |
| Male, n | 48 (75%) | 352 (71%) | 71 (74%) | 0.458 | 0.882 | 0.471 |
| Age, years | 42.4±2.1 | 40.9±0.7 | 49.3±0.9 | 0.452 | 0.005 | ＜0.001 |
| Body weight, kg | 55.6±1.4 | 54.7±0.5 | 57.0±1.0 | 0.539 | 0.891 | 0.054 |
| Time between symptoms and admission, months | 0.54±0.04 | 2.7±0.2 | 1.65±0.1 | ＜0.001 | ＜0.001 | ＜0.001 |
| Vegetation length, mm | 5.0±0.2 | 10.4±0.9 | 13.83±0.33 | ＜0.001 | ＜0.001 | ＜0.001 |
