# Supplementary material for: Results of the inoperable and operable with aortic valve endocarditis
Source: Front Cardiovasc Med. 2024 Jan 16;10:1296557. doi: 10.3389/fcvm.2023.1296557 (PMC10824924; doi:10.3389/fcvm.2023.1296557)
Supplement: Supplementary file 4 [file Table4.docx]

Table 4. Preoperative, Operative and follow-up data (n=512)

| Variable | *Total*  *(n=512)* | Group with destruction of the aortic annulus  (n=80) | Group without destruction of the aortic annulus  (n=432) | P value |
| --- | --- | --- | --- | --- |
| Preoperative |  |  |  |  |
| Male, n (%) |  | (%) | (%) |  |
| Age, years | *40.63±0.69* | 39.8±1.2 | 40.78±0.78 | 0.605 |
| Weight, kg | *54.69±0.52* | 62.6±0.58 | 53.22±0.58 | ＜0.001 |
| Time between symptoms and surgery，months | *2.64±0.11* | 2.52±0.28 | 2.66±0.12 | 0.638 |
| Vegetation length, mm | *10.75±0.3* | 13.2±0.48 | 10.3±0.33 | ＜0.001 |
| Preoperative left ventricular end diastolic dimension, mm | *64.03±0.38* | 70.6±0.67 | 62.77±0.40 | ＜0.001 |
| Preoperative left ventricular ejection fractions, % | *60.32±0.37* | 59.0±0.81 | 60.58±0.41 | 0.114 |
| Preoperative aortic insufficiency, cm^2^ | *9.02±0.28* | 12.20±0.30 | 8.43±0.32 | ＜0.001 |
| Serum creatinine before surgery, μmol/L | *84.13±1.26* | 105.6±3.92 | 80.15±1.21 | ＜0.001 |
| Operative |  |  |  |  |
| In-hospital mortality, n | *32（6.3%）* | 18（22.5%） | 14 （3.2%） | ＜0.001 |
| Aortic cross-clamp time, minutes | *97.81±1.56* | 122.8±3.56 | 93.19±1.63 | ＜0.001 |
| Cardiopulmonary bypass time, minutes | *155.34±2.27* | 209.8±5.80 | 145.3±2.15 | ＜0.001 |
| Mechanical ventilation time, hours | *55.23±2.42* | 73.2±5.66 | 51.91±2.64 | 0.001 |
| ICU retention time, days | *5.47±0.13* | 7.0±0.19 | 5.19±0.15 | ＜0.001 |
| Hospitalized time postoperative, days | *19.47±0.38* | 21.2±1.04 | 19.15±0.4 | 0.047 |
| Serum creatinine 24h after surgery, μmol/L | *98.59±1.96* | 134.8±7.27 | 91.89±1.72 | ＜0.001 |
| Serum creatinine 48h after surgery, μmol/L | *116.94±3.06* | 150.2±11.57 | 110.78±2.84 | ＜0.001 |
| Fluid balance on operation day, ml | *-586.88±36.21* | -600.0±48.78 | -584.4±42.0 | 0.876 |
| Fluid balance on 1st day postoperative, ml | *-637.50±54.06* | -440.0±63.19 | -674.1±62.85 | 0.116 |
| Fluid balance on 2nd day postoperative, ml | *-446.88±34.48* | -200.0±31.82 | -492.6±40.1 | 0.002 |
| Chest drainage, ml | *686.88±15.41* | 786.0±41.14 | 668.5±16.48 | 0.006 |
| Postoperative left ventricular end diastolic dimension, mm | *49.61±0.32* | 55.0±0.64 | 48.58±0.34 | ＜0.001 |
| Postoperative left ventricular ejection fractions, % | *58.15±0.34* | 55.4±0.78 | 58.68±0.37 | ＜0.001 |
| Fresh-frozen plasma, ml | *722.81±17.94* | 930.0±36.53 | 684.4±19.62 | ＜0.001 |
| Packed red cells, units | *2.91±0.12* | 4.20±0.35 | 2.67±0.12 | ＜0.001 |
| Follow-up | *Total*  *(n=460)* | Group with destruction of the aortic annulus  (n=60) | Group without destruction of the aortic annulus  (n=400) | P value |
| Length of follow-up, months | *74.96 ± 2.44* | 36.5±5.6 | 77.8±2.6 | ＜0.001 |
| All-time mortality, n | *79(17.2%)* | 42(70%) | 37(9.3%) | ＜0.001 |
| All-time re-operation, n | *41(8.9%)* | 18(30%) | 23(5.8%) | ＜0.001 |

ICU= intensive care unit
