# Supplementary material for: Results of the inoperable and operable with aortic valve endocarditis
Source: Front Cardiovasc Med. 2024 Jan 16;10:1296557. doi: 10.3389/fcvm.2023.1296557 (PMC10824924; doi:10.3389/fcvm.2023.1296557)
Supplement: Supplementary file 5 [file Table5.docx]

Table 5. Operation in aortic valve endocarditis (n=512)

| Variable | Value |
| --- | --- |
| Operation |  |
| Isolated aortic valve replacement, n | 176/512(34.4%) |
| Double valve operation, n | 320/512(62.5%) |
| Bentall + mitral valve replacement, n | 16/512(3.1%) |
| No root operation | 432/512(84.4%) |
| Aortic root operation | 80(15.6%) |
| 6.0 Prolene sutures | 15/80(18.75%) |
| Patch reconstruction | 49/80(61.25%) |
| Aortic root replacement | 16/80(20%) |
