# Supplementary material for: Results of the inoperable and operable with aortic valve endocarditis
Source: Front Cardiovasc Med. 2024 Jan 16;10:1296557. doi: 10.3389/fcvm.2023.1296557 (PMC10824924; doi:10.3389/fcvm.2023.1296557)
Supplement: Supplementary file 6 [file Table6.docx]

Table 6. Analysis of risk factors for destruction of the aortic annulus in aortic valve endocarditis

| Model | OR | 95% CI | P value |
| --- | --- | --- | --- |
| Univariate analysis | | | |
| Body weight | 0.898 | 0.868-0.928 | ＜0.001 |
| Vegetation length | 0.942 | 0.911-0.974 | ＜0.001 |
| Preoperative aortic insufficiency | 0.919 | 0.888-0.952 | ＜0.001 |
| Preoperative left ventricular end diastolic dimension | 0.900 | 0.874-0.926 | ＜0.001 |
| Serum creatinine before surgery | 0.970 | 0.962-0.979 | ＜0.001 |
| Multivariate analysis | | | |
| Body weight | 0.897 | 0.862-0.933 | ＜0.001 |
| Vegetation length | 0.926 | 0.887-0.967 | 0.001 |
| Preoperative aortic regurgitation | 0.934 | 0.897-0.972 | 0.001 |
| Preoperative left ventricular end diastolic dimension | 0.928 | 0.897-0.959 | ＜0.001 |
| Serum creatinine before surgery | 0.976 | 0.966-0.987 | ＜0.001 |
