# Supplementary material for: Results of the inoperable and operable with aortic valve endocarditis
Source: Front Cardiovasc Med. 2024 Jan 16;10:1296557. doi: 10.3389/fcvm.2023.1296557 (PMC10824924; doi:10.3389/fcvm.2023.1296557)
Supplement: Supplementary file 7 [file Table7.docx]

Table 7. Analysis of the significance of destruction of the aortic annulus in aortic valve endocarditis (n=512)

| Model | OR | 95% CI | P value |
| --- | --- | --- | --- |
| Univariate analysis of risk factors for in-hospital mortality following cardiac surgery (n=32) | | | |
| Destruction of the aortic annulus | 6.500 | 3.097-13.641 | ＜0.001 |
| Multivariate analysis of risk factors for in-hospital mortality following cardiac surgery (n=32) | | | |
| Destruction of the aortic annulus | 5.847 | 2.767-12.355 | ＜0.001 |
| Univariate analysis of risk factors for prolonged mechanical ventilation time (mechanical ventilation time> 96h, n=128) following cardiac surgery | | | |
| Destruction of the aortic annulus | 2.333 | 1.413-3.853 | 0.001 |
| Multivariate analysis of risk factors for prolonged mechanical ventilation time (mechanical ventilation time> 96h, n=128) following cardiac surgery | | | |
| Destruction of the aortic annulus | 1.890 | 1.117-3.917 | 0.018 |
| Univariate analysis of risk factors for prolonged ICU retention time (ICU retention time > 7d, n=176) following cardiac surgery | | | |
| Destruction of the aortic annulus | 11.429 | 6.344-20.588 | ＜0.001 |
| Multivariate analysis of risk factors for prolonged ICU retention time (ICU retention time > 7d, n=176) following cardiac surgery | | | |
| Destruction of the aortic annulus | 9.956 | 5.413-18.313 | ＜0.001 |
| Univariate analysis of risk factors for early aortic paravalvular leak following cardiac surgery (n=28) | | | |
| Destruction of the aortic annulus | 7.448 | 4.228-12.936 | ＜0.001 |
| Multivariate analysis of risk factors of early aortic paravalvular leak following cardiac surgery (n=28) | | | |
| Destruction of the aortic annulus | 7.183 | 4.036-12.785 | ＜0.001 |
| Univariate analysis of risk factors for 1-year mortality following cardiac surgery (n=46) | | | |
| Destruction of the aortic annulus | 25.467 | 12.502-51.877 | ＜0.001 |
| Multivariate analysis of risk factors for 1-year mortality following cardiac surgery (n=46) | | | |
| Destruction of the aortic annulus | 43.316 | 12.77-146.91 | ＜0.001 |

ICU= intensive care unit
