# Supplementary material for: Results of the inoperable and operable with aortic valve endocarditis
Source: Front Cardiovasc Med. 2024 Jan 16;10:1296557. doi: 10.3389/fcvm.2023.1296557 (PMC10824924; doi:10.3389/fcvm.2023.1296557)
Supplement: Supplementary file 8 [file Table8.docx]

Table 8. Cox proportional hazard regression for all-time mortality

| Model | OR | 95% CI | P value |
| --- | --- | --- | --- |
| Univariate analysis | | | |
| Male | 0.026 | 0.001-0.542 | 0.018 |
| Age | 0.951 | 0.924-0.979 | 0.001 |
| Body weight | 1.055 | 1.028-1.083 | ＜0.001 |
| Vegetation length | 1.085 | 1.040-1.131 | ＜0.001 |
| Preoperative aortic insufficiency | 1.041 | 1.004-1.079 | 0.029 |
| ICU retention time | 1.699 | 1.455-1.983 | ＜0.001 |
| Serum creatinine 48h after surgery | 1.301 | 1.170-1.458 | ＜0.001 |
| Postoperative left ventricular end diastolic dimension | 1.041 | 1.010-1.073 | 0.004 |
| Packed red cells | 1.330 | 1.218-1.453 | ＜0.001 |
| Multivariate analysis | | | |
| Age | 0.938 | 0.905-0.973 | 0.001 |
| Body weight | 1.043 | 1.020-1.067 | ＜0.001 |
| Vegetation length | 1.407 | 1.226-1.616 | ＜0.001 |
| Preoperative aortic insufficiency | 0.888 | 0.829-0.951 | 0.001 |
| ICU retention time | 1.477 | 1.214-1.798 | ＜0.001 |
| Serum creatinine 48h after surgery | 1.290 | 1.159-1.338 | ＜0.001 |
| Postoperative left ventricular end diastolic dimension | 1.309 | 1.173-1.462 | ＜0.001 |
| Packed red cells | 1.830 | 1.540-2.174 | ＜0.001 |

ICU= intensive care unit
